# The Chemical Composition and Nitrogen Distribution of Chinese Yak (Maiwa) Milk

**DOI:** 10.3390/ijms12084885

**Published:** 2011-08-02

**Authors:** Haimei Li, Ying Ma, Qiming Li, Jiaqi Wang, Jinju Cheng, Jun Xue, John Shi

**Affiliations:** 1 School of Food Science and Engineering, Harbin Institute of Technology, Harbin 150090, China; E-Mails: haimeili@ht.edu.cn (H.L.); chengjinju1987@163.com (J.C.); 2 Research and Development Central of New Hope Dairy Holding Company, Chengdu 610041, China; E-Mails: qm258@126.com (Q.L.); wjq722006@126.com (J.W.); 3 Guelph Food Research Center, Agriculture and Agri-Food Canada, 1341 Baseline Road, Ottawa, Ontario, K1A 0C5, Canada; E-Mails: Jun.Xue@agr.gc.ca (J.X.); John.Shi@agr.gc.ca (J.S.)

**Keywords:** chemical composition, nitrogen distribution, seasonal changes, yak milk

## Abstract

The paper surveyed the chemical composition and nitrogen distribution of Maiwa yak milk, and compared the results with reference composition of cow milk. Compared to cow milk, yak milk was richer in protein (especially whey protein), essential amino acids, fat, lactose and minerals (except phosphorus). The contents of some nutrients (total protein, lactose, essential amino acids and casein) were higher in the warm season than in the cold season. Higher ratios of total essential amino acids/total amino acids (TEAA/TAA) and total essential amino acids/total non essential amino acids (TEAA/TNEAA) were found in the yak milk from the warm season. However its annual average ratio of EAA/TAA and that of EAA/NEAA were similar to those of cow milk. Yak milk was rich in calcium and iron (p < 0.05), and thus may serve as a nutritional ingredient with a potential application in industrial processing.

## 1. Introduction

Yaks are found extensively on the plateau of the western Tibetan region of China in alpine and subalpine areas at altitudes from 2000–5000 m with a cold, semi-humid climate [1]. Domestic yak milk and yak meat is a vital part of the local economy in the Tibetan region of China. Yak milk is called natural concentrated milk because of its high fat (5.5–7.5%), protein (4.0–5.9%) and lactose (4.0–5.9%) content during the main lactating period [2,3]. Yak milk and milk products are the major ingredients of the daily diet of Tibetan herders, particularly for the weak, ill, elderly and young in the areas where yaks graze on the alpine meadows and mountain pastures. Due to a shortage of fruit and vegetables, and limited food resources, yak milk and milk products (butter and cheese) are a vital source of vitamins and major sources of nutrition for Tibetan Herders [4]. However, large-scale industrialization of yak milk production is limited by low volume and seasonal cyclicity of individual milk production, around 150–500 kg of fresh milk per lactation, which depends on the breed, age, parity and body condition of the yak, pasture growth pasture quality, raising areas, milking time, milking methods, and other environmental factors [1,4].

In recent years, production of yak milk has seen an increase to 40 million tons each year. However, industrially processed dairy products were no more than 25%, while the remainder was produced and consumed by traditional means [5]. Yak milk and milk products are gaining in popularity due to their special nutritional value. In China, Several dairy companies are being established to supply fresh pasteurized and UHT milk to consumers. Considering its economic potential, information on the composition as well as the chemical properties of yak milk is essential for the successful development of a yak product industry and for marketing.

There are differences in chemical characteristics between yak and cow milk. Yak milk is predominantly produced by seasonal breeding. Its composition varies with seasonal grass growth and climate change, as does milk production. The highest content of the composition, such as solids, lactose, protein and amino acids, are in the mid-lactation period but the fat content increases continuously into late lactation [6,7]. On the other hand, the bulk milk from cow herds varies little with the seasons because of year-round breeding, therefore the composition of cow’s milk shows minimal changes throughout the year [8].

In China, According to the (Chinese) provincial annals of livestock breeds, there are 12 officially recognized breeds of domestic yak in China: the Jiulong yak and Maiwa yak in Sichuan province, Tianzhu White yak and Gannan yak in Gansu province, Pali yak, Jiali (“Alpine”) yak and Sibu yak in Tibet, Huanhu yak and Plateau yak in Qinghai province, Bazhou yak in Xinjiang and Zhongdian yak in Yunnan province, and one other, the “Long-hair-forehead yak” in Qinghai province. These 12 yak breeds belong to two main types, the Qinghai-Tibet Plateau type ('Plateau or Grassland type) and the Henduan Alpine type (Alpine or Valley type) [1]. Maiwa yak belongs to the Qinghai-Tibet Plateau type, numbering around 5.4 million animals, and ranking second in China’s yak population [5]. Yak and yak milk have come to occupy a dominant position in the local economy.

Cow milk is a complex colloidal dispersion containing fat globules, casein micelles and whey proteins in an aqueous solution of lactose, minerals and a few other minor compounds. Its chemical properties depend on intrinsic compositional and structural factors, and on extrinsic factors such as temperature and post–milking treatments. Although the composition of yak milk has been studied in China [2,3,6,7,9], there is little information on the chemical composition, nitrogen distribution and mineral contents of yak milk produced from Maiwa breeds in the Qinghai-Tibet Plateau of China. The purpose of this paper is to investigate the chemical properties of yak milk from the Maiwa breed and to compare variation of these parameters in cold and warm season. The results were compared with those obtained from cow’s milk. An understanding of these properties is important in the elucidation of complex chemical reactions that occur in yak milk and in the technological and engineering design and operation of the milk processes and processing equipment.

## 2. Results and Discussion

### 2.1. Basic Chemical Composition of Yak Milk

The basic composition (fat, protein, lactose, ash and total solids) of yak milk is shown in Table 1. Except for ash content, the average content of fat, protein, lactose and total solid of the yak milk is higher than cow milk from the USA and the surrounding area [3,10]. The fat content of the yak milk was higher in the cold season than in the warm season, while the contents of total protein and lactose were higher in the warm season (p < 0.05). In general, the current data is similar to the proximate composition of other yak breeds published so far [1,11,12]. Because yak are allowed to graze under uncontrolled environmental conditions, the milk composition varies with seasonal grass growth and climate changes [11,12]. On the Qinghai-Tibet Plateau, the average annual air temperature is generally bellow 0 °C, while the average temperature in January drops below −10 °C. The average temperature in the hottest month (July) does not exceed 13 °C. During the warm season, the plentiful green grass is enough to feed yaks. The cold pastures on which the yak were grazed have predominantly short grass and rough grazing conditions, with sedges and shrubby plants [1]. With the growth of forage (from August to December), protein content in the swards declines from 115 g per kg DM (young grass) to 33 g per kg DM (mature grass), and crude fiber in the swards increases correspondingly [1,12]. The increased crude fiber can offer more acetic acid and butyric acid (the sources of fatty acids) for the mammary gland to synthesize more fat which may be the reason for the higher fat content of yak milk in the cold season [1]. The fat content in ovine milk showed similar seasonal changes from February to August (7.58% to 6.59%) [13].

The protein content of Maiwa yak was far higher than that of other dairy breeds, but similar to that of buffalo milk [14]. The protein content of Maiwa yak was similar to that of yak breeds of Jiulong (4.9%), Tianzhu white (5.2%) and Jiali (5.0%), but lower than that of the yak breeds, like Pali (5.7%), Kyrgyzstan (5.3%), India (5.9%) and Nepa (5.4%) [1]. Sheng *et al.* [9] surveyed the protein content of Maiwa yak milk which was collected in October from the seven mid-lactating yaks. The protein content (3.51%) seemed to be low compared with the average value (4.95%) of the milk from whole year. In this study, the average protein content of the milk was within the range of reported data (4.0–5.5%) [1].

The lactose content of Maiwa yak milk was 5.03% higher than that of cow milk. Higher contents of lactose in the yak milk will be beneficial to infants. Lactose in the distal bowel can help combat gastrointestinal disturbances resulting from undesirable putrefactive bacteria through promoting the growth of certain beneficial lactic-acid-producing bacteria [15].

The ash content of the yak milk (0.79%) was similar to that of cow milk. In general, ash content of yak milk is around 0.7–0.9% during main lactating period. Furthermore, Weiner *et al.* [1] and Jiang *et al.* [11] analyzed the ash contents of Maiwa yak milk from June to September, and the ash content (0.82 ± 0.06%) did not show significant changes with season.

### 2.2. Nitrogen Distribution in Yak Milk

The N-containing portions of milk can be divided into three broad fractions, including casein nitrogen (CN), whey protein nitrogen (WPN), and non protein nitrogen (NPN). Variations in the differences in nitrogen distribution of yak milk proteins between warm and cold seasons are shown in Table 2. Total nitrogen (TN) of yak milk from the warm season was higher than that from the cold season (p < 0.05). The protein contents may be affected by several factors, including breed, environmental temperature, diseases, and stage of lactation, parity and nutrition [16]. In general, high environmental temperature reduces the total protein content of milk [17], so the protein content of cow milk was higher during winters than during summers [16]. However, several papers have reported that energy intake (except fats and oils) may have a positive effect on milk protein content [18–20]. In the Qinghai-Tibet Plateau, grass and herbs cannot survive in a very cold winter. For natural grazing yaks, the malnutrition of feeds resulted in lower TN in winter. Similarly, Walley *et al.* [21] demonstrated that the total protein content of milk was lowest when the energy intake of the cow was retarded.

The NPN contents of the yak milk were 0.05% and 0.04% respectively in warm and cold seasons. The NPN constituted 5.56% and 5.30% of the total N in the yak milk, which did not vary much between seasons; and that the NPN proportion was consistent with that of cow’s milk [16]. The reason of above results is that the NPN content of milk is less variable among breeds, and the changes of milk NPN content with environmental temperature are similar in pattern to changes in protein content [16].

The contents of the whey protein nitrogen (WPN) did not change significantly between cold and warm seasons. The average WPN/TN of the yak milk was 19.63% which was higher than that of cow milk [16], but it was similar to that of sheep milk [22]. The average value of α-lactalbumin and β-lactoglobulin as a percentage of whey protein was upwards of 66% which was lower than that of cow whey protein [23]. The value found for α-lactalbumin is puzzling because it is not in line with the usual relationship between lactose and α-lactalbumin contents in mammalian milks. Indeed, α-lactalbumin is half of the lactose synthetase enzyme and its content increases with lactose concentration [24]. In the collected samples of yak milk, the lactose concentrations were higher than that of bovine milk. However, the study on yak milk proteins has highlighted the lack of knowledge in the literature; deep further study is required.

Although the level of CN was different between the warm and cold seasons (p < 0.05), the ratio of CN/TN was not significantly different. The highest level of CN was found in the warm season, which was closely consistent with the changes of TN [23,25]. For yak milk, the variation patterns of CN and CN/TN were consistent with those found in North American commingled goat milk [25]. The TN and CN showed significant variability between warm and cold seasons, while the ratios of nitrogen distribution, such as NPN/TN, WPN/TN, WPN/CN and CN/TN, remained constant throughout the seasons. Therefore, this pattern of nitrogen distribution might be useful in deciding the end use for yak milk.

The analysis results of amino acids, such as the contents of amino acids, total essential amino acids (TEAA), total non essential amino acids (TNEAA), and total amino acids (TAA), are given in Table 3. The results showed that the contents of some amino acids were unaffected by seasons. In the warm season, the TEAA was higher than that in the cold season (p < 0.05) which was consistent with the changes of the total protein content (Table 1); however, the TNEAA was not significantly different in the warm and cold seasons. In the warm season, the ratios of TEAA/TNEAA and TEAA/TAA were significantly higher than those in cold seasons, while the yearly average ratios of TEAA/TNEAA and TEAA/TAA were similar to those of cow milk [26]. The ratio of TEAA/TNEAA was attributed to the protein ratio of herbage grazed by the yaks [27]. Because the yaks were allowed to graze naturally at an average elevation of 3600 m on the Qinghai-Tibet Plateau, the important factors affecting milk quality are: pasture production and the quantity, growth status and nutritive value of the herbage. This means that all lactating yaks, irrespective of age, parity or breed type, or even location, tended to peak in yield in the summer season (June to August) when grass was at its best quality and quantity, while after August, as air temperature fell, the nutritive value declined [1]. Therefore, the TN, CN and TEAA of the yak milk were lower in the cold season.

### 2.3. Mineral Contents of Yak Milk

The major mineral contents of the yak milk from cold and warm seasons are given in Table 4. Yak milk and cow milk had similar ash content, around 0.8%. The major mineral contents of yak milk were much higher than those of cow milk while the content of phosphorous was in the range of cow’s milk [28]. The contents of these minerals did not show significant differences between warm and cold seasons, which kept the same trend as ash content. The average calcium content of the yak milk is 1545.45 mg/kg, while human milk has only one-fifth the amount of this mineral [22]. Dietary iron is required for a wide variety of biochemical processes. Human milk, as well as bovine milk and milk products, are poor sources of iron. To prevent iron deficiency and anemia in infants of 6–9 months, most infant formulas are supplemented with iron [29]. So the higher iron content (0.57 mg/kg) of yak milk can be a benefit in its nutritional value in infant foods. In general, the mineral content of yak milk seems to vary much more than that of cow milk due to the monthly differences in feeding. The trace minerals in yak milk have not been extensively studied, even though they may be of considerable nutritional and health interest to humans.

## 3. Experimental Section

### 3.1. Collection of Milk Samples

Sichuan province is the largest yak-raising province in China, there is more than four million yak and yak hybrids in the western and northern parts of Sichuan. Yak, in western parts of Sichuan, are found in all counties in Ganzi Tibetan autonomous prefecture and in Aba Tibetan and Qiang autonomous prefecture in the northeastern end of western Sichuan and most of counties in Liangshan Yi autonomous prefecture in the southern part of western Sichuan. Yak contributes 70 percent of the total milk production (180,000 tons annually) in Sichuan, much of it from Ganzi (Jiulong breed of yak) and Aba (Maiwa breed of yak). Numbers of yak have increased more in Aba than in Ganzi, most likely because of a better access to markets for yak products in Aba. Therefore the Maiwa breed of yak was selected for study.

One hundred and four pure Maiwa milk samples, in west part of Sichuan province, were collected monthly from Hongyuan County (31°51’–33°19’ N, 101°51’–103°23’ E) in May to December of 2009. All of the yak milk samples were collected from a family pasture. All of the yaks under study were grazing on natural pasture and did not have any supplementary feeding. About 20 percent of herds were less than 5 years old, with 2 parities. In the warm season (from May to September), yak was grazed in summer-autumn pasture where the altitude is about 4000 m, and in the cold season (from end of October to next April), yak was grazed in winter-spring pasture where the altitude is about 3200 m. 56 yak milk samples were collected in the warm season and 48 yak milk samples were collected in the cold season. The fresh milk samples were collected in sterile plastic bottles, and immediately were transported to the nearest town by car. The milk samples were frozen in a −18 °C refrigerator. The frozen milk samples were transported to the laboratory by airplane. In the laboratory, the frozen milk samples were thawed and their chemical parameters were analyzed. The extra milk samples were stored at −20 °C. The entire time from milking to beginning analysis was about 60 h.

### 3.2. The Determination of Basic Compositions

The frozen yak milk samples were thawed to 38 ± 1 °C. The samples were gently thoroughly mixed by repeatedly inverting the sample bottle without causing frothing or churning. They were cooled to room temperature immediately before the analyses. The contents of total nitrogen (TN) and nonprotein nitrogen (NPN) were tested by Kjeldahl method according to the IDF 020-1 [30] and IDF 020-4 [31]. Non casein nitrogen (NCN) was tested according to describe by Wehr *et al.* [32]. Casein was precipitated by acetic acid, NCN in the filtrate was detected by the Kjeldahl method. The nitrogen fraction were calculated as follows: protein nitrogen (PN) = TN-NPN, casein nitrogen (CN) = TN-NCN, and whey protein nitrogen = NCN-NPN. A nitrogen conversion factor of 6.38 was used to calculate protein contents of milk samples and various fractions. The fat content was tested with gravimetric method according to IDF 001D [33]. Lactose was tested according to IDF 028A [34]. Ash content was tested after mineralisation of milk at 550 °C for 4 h according to IDF 027 [35]. The total solid (TS) was tested by drying 5 grams of milk sample at 100 ± 2 °C for 5 h in porcelain crucibles according to IDF 021B [36]. Beta-lactoglobulin and alfa-lactalbumin were measured according to Bordin *et al.* (2001) [37]. Standard bovine whey proteins were purchased from Sigma. 10 mg α-La (lot L-5385 type I, ∼85%), 15 mg β-LgB (lot L-8005) and 10 mg β-LgA (lot L-7880) were dissolved in 5ml buffer solution (8M urea, 165 mM Tris, 44 mM sodium citrate and 0.3% (v/v) β-mercaptoethanol). 1 mL of skimmed yak milk was dissolved in 4 mL of buffer solution. The diluted samples were filtered through a 0.45 μm cellulose membrane and directly analyzed.

### 3.3. The Analysis of Amino Acids

1 mL of milk samples were hydrolyzed with 6.0 M HCl in vacuum-sealed tubes at 110 °C for 22 h. The amino acids content was tested using an amino acid analyzer (L-8800, Hitachi, Japan). Amino acids were separated on a single-column ion-exchange chromatograph (Hitachi, 2622#, 4.6 mm × 60 mm) and were post-column derived with ninhydrin. Derivative amino acids were analyzed by spectrophotometer (570 nm and 440 nm) and quantified by comparing the area under the sample peak against that of an amino acid standard solution (Hitachi, Japan) of known content. The detailed procedure followed which was described by Zhang *et al.* [38]. Tryptophan was lost during hydrolysis; therefore, tryptophan values are not reported. The results were given as means of triplicate analyses.

### 3.4. Mineral Assay of Samples

The contents of calcium, magnesium, copper, iron, manganese and zinc were analyzed by an Atomic Absorption Spectrometer (Atomic Spectormetry Analyst 800, Perkin Elmer, Vernon Hills, IL, USA) according to the standard method of GB 5413.21 [39]. The P content was tested by Spectrophotometer (WFZ754, Shanghai, China) according to IDF042 [40]. Each sample was analyzed in triplicate.

### 3.5. Statistical Analysis

Data were analyzed by ANOVA method using SPSS software, version 10.0 (SPSS, Inc., Chicago, IL, USA). The differences among the means of the analysis data were compared at a significance level of p < 0.05.

## 4. Conclusions

This work showed that the composition of most of the analyzed yak milk was affected by seasons, and differed in some aspects from cow’s milk. The yak milk has higher contents of fat, protein, lactose, and total solids than cow milk, and its contents changed with the seasons. The yak milk protein contained a higher ratio of WPN/TN. It is noteworthy that calcium and iron were richer in yak milk than in cow milk. These differences suggested that yak milk can serve as nutritional ingredients with the potential for industrial processing. On the other hand, it will be interesting to further test yak milk properties, like acidification and heat stability. The knowledge will benefit the milk processing industry.

## Figures and Tables

**Table 1.** The basic chemical compositions of yak milk (g/100 g fresh milk).

	**Warm season (n = 56)**	**Cold season (n = 48)**	**Average**	Cow milk *
Fat	5.04 ± 0.46 ^a^	6.84 ± 0.53 ^b^	6.12 ± 0.60	2.4–5.5
Total Protein	5.30 ± 0.25 ^a^	4.72 ± 0.34 ^b^	4.95 ± 0.53	2.3–4.4
Lactose	5.50 ± 0.32 ^a^	4.77 ± 0.38 ^b^	5.03 ± 0.43	3.8–5.3
Ash	0.82 ± 0.06 ^a^	0.76 ± 0.05 ^a^	0.79 ± 0.05	0.68–0.80
Total Solids	16.66 ± 1.26 ^a^	17.09 ± 1.38 ^b^	16.88 ± 1.36	11.3–14.5

* Reference [10];

both ^a^ and ^b^ occurring in the same line means that the results are significantly different (p < 0.05); n: the number of samples analyzed and computed in the statistical analysis.

**Table 2.** Concentration (%) of the nitrogen containing fractions in yak milk.

	**Warm season (n = 56)**	**Cold season (n = 48)**	**Average**	Cow milk*
TN	0.83 ± 0.05 ^a^	0.74 ± 0.03 ^b^	0.79 ± 0.04	0.36–0.69
NPN	0.05 ± 0.01 ^a^	0.04 ±0.01 ^a^	0.04 ± 0.01	0.023–0.042(0.03)
NPN/TN	5.56 ± 0.25	5.30 ± 0.24	5.50 ± 0.32	∼5
WPN	0.16 ± 0.01 ^a^	0.15 ± 0.01 ^a^	0.15 ± 0.01	
β-lactoglobulin	0.60 ± 0.04 ^a^	0.58 ± 0.04 ^a^	0.59 ± 0.05	0.33
α-lactalbumin	0.07 ± 0.01 ^a^	0.04 ± 0.01 ^b^	0.05 ± 0.01	0.12
WPN/TN	19.01 ± 1.78	20.10 ± 1.89	19.63 ± 2.03	17
CN	0.63 ± 0.003 ^a^	0.55 ± 0.02 ^b^	0.60 ± 0.03	
CN/TN	75.41 ± 2.22	74.00 ± 5.21	74.63 ± 4.18	78
WPN/CN	25.30 ± 1.65	27.60 ± 1.75	26.56 ± 1.69	22

* Reference [10];

both ^a^ and ^b^ occurring in the same line means that the resultsare significantly different (p < 0.05); n: the number of samples analyzed and computed in the statistical analysis.

**Table 3.** Amino acid contents of yak milk (g/100 g).

	**Warm season N = (56)**	**Cold season N = (48)**	**Average**	Cow milk*
Essential Amino-Acid (EAA)				
Thr	0.21 ± 0.01 ^a^	0.18 ± 0.02 ^b^	0.19 ± 0.02	0.15
Val	0.30 ± 0.03 ^a^	0.24 ± 0.03 ^b^	0.26 ± 0.04	0.16
Met	0.12 ± 0.01 ^a^	0.11 ± 0.01 ^a^	0.11 ± 0.01	0.06
Ile	0.27 ± 0.03 ^a^	0.22 ± 0.03 ^b^	0.24 ± 0.04	0.14
Leu	0.46 ± 0.03 ^a^	0.41 ± 0.04 ^b^	0.43 ± 0.04	0.29
Phe	0.23 ± 0.01 ^a^	0.21 ± 0.02 ^a^	0.22 ± 0.02	0.16
Lys	0.43 ± 0.04 ^a^	0.36 ± 0.04 ^b^	0.38 ± 0.06	0.27
His	0.13 ± 0.01 ^a^	0.11 ± 0.01 ^b^	0.12 ± 0.01	0.1
Trp	ND	ND	ND	0.05
TEAA	2.16 ± 0.22 ^a^	1.84 ± 0.24 ^b^	1.95 ± 0.24	1.33
Non-Essential Amino Acid (NEAA)				
Cys	0.04 ± 0.01 ^a^	0.03 ± 0.01 ^a^	0.04 ± 0.01	0.02
Arg	0.17 ± 0.01 ^a^	0.15 ± 0.02 ^b^	0.16 ± 0.02	0.11
Pro	0.44 ± 0.02 ^a^	0.47 ± 0.06 ^a^	0.46 ± 0.05	0.32
Asp	0.37 ± 0.03 ^a^	0.31 ± 0.03 ^b^	0.33 ± 0.04	0.26
Ser	0.24 ± 0.01 ^a^	0.23 ± 0.02 ^a^	0.23 ± 0.02	0.16
Glu	1.14 ± 0.08 ^a^	1.00 ± 0.10 ^b^	1.05 ± 0.12	0.77
Gly	0.11 ± 0.02 ^a^	0.16 ± 0.09 ^b^	0.12 ± 0.10	0.06
Ala	0.13 ± 0.01 ^a^	0.14 ± 0.02 ^a^	0.14 ± 0.02	0.1
Tyr	0.23 ± 0.02 ^a^	0.21 ± 0.02 ^a^	0.22 ± 0.02	0.15
TNEAA	2.87 ± 0.15 ^a^	2.70 ± 0.21 ^a^	2.72 ± 0.22	1.95
TAA	5.04 ± 0.29 ^a^	4.53 ± 0.42 ^a^	4.67 ± 0.42	3.33
TEAA/TNEAA	0.75 ± 0.03 ^a^	0.68 ± 0.03 ^b^	0.72 ± 0.05	0.68
TEAA/TAA	0.43 ± 0.01 ^a^	0.41 ± 0.01 ^b^	0.42 ± 0.02	0.40

* Reference [26];

both ^a^ and ^b^ occurring in the same line means that the results are significantly different (p < 0.05); n: the number of samples analyzed and computed in the statistical analysis.

**Table 4.** The mineral contents in yak milk (mg/kg).

	**Warm season**	**Cold season**	**Average**	Cow milk*
Cu	0.42 ± 0.06 ^a^	1.44 ± 0.07 ^b^	1.07 ± 0.08	0.1–0.6
Mg	154.37 ± 13.77 ^a^	153.96 ± 12.32 ^a^	154.10 ± 13.22	90.00–140.00
Zn	8.03 ± 0.52 ^a^	6.93 ± 0.33 ^a^	7.31 ± 0.44	2.00–6.00
Fe	0.40 ± 0.03 ^a^	0.65 ± 0.03 ^b^	0.57 ± 0.04	0.16–0.35
Mn	0.06 ± 0.01 ^a^	0.04 ± 0.01 ^a^	0.06 ± 0.01	0.012–0.035
Ca	1524.52 ± 138.86 ^a^	1556.49 ± 193.17 ^a^	1545.45 ± 145.61	1000.00–1300.00
P	902.96 ± 58.99 ^a^	944.93 ± 81.12 ^a^	922.04 ± 70.13	900.00–1000.00

* Reference [28];

both ^a^ and ^b^ occurring in the same line means that the results are significantly different (p < 0.05).
